# Safety and Immunogenicity of Live Oral Cholera Vaccine CVD 103-HgR in Children and Adolescents Aged 6–17 Years

**DOI:** 10.4269/ajtmh.19-0241

**Published:** 2019-11-25

**Authors:** James M. McCarty, Emma C. Gierman, Lisa Bedell, Michael D. Lock, Sean Bennett

**Affiliations:** 1 Stanford University School of Medicine, Stanford, California;; 2 PaxVax, Inc., Redwood City, California

## Abstract

The attenuated recombinant *Vibrio cholerae* O1 vaccine strain CVD 103-HgR, redeveloped as PXVX0200, elicits a rapid serum vibriocidal antibody (SVA) response and protects against cholera-induced diarrhea in adult volunteer challenge trials but has not been studied in children in developed countries. We performed a phase 4, placebo-controlled, double-blind, multicenter study to assess the safety, immunogenicity, and tolerability of a single, oral dose of PXVX0200 in children and adolescents aged 6–17 years in the United States and bridged immunogenicity to adults aged 18–45 years from a separate lot consistency study. Volunteers were randomized to receive a single dose of 1 × 10^9^ colony forming units (CFU) of PXVX0200 or placebo. Immunogenicity endpoints included SVA levels on days 1, 11, and 29 in volunteers aged 6–17 years and also on days 91 and 181 in volunteers aged 12–17 years. Safety was assessed by comparing solicited signs and symptoms on days 1–8, unsolicited adverse events (AEs) through day 29, and serious AEs through day 181. A total of 374 participants were enrolled, comprising 321 vaccine and 53 placebo recipients. The SVA seroconversion rates 10 days after immunization were 98.6% and 2.1% in vaccine and placebo recipients, respectively, and the vaccine seroconversion rate was non-inferior to the 93.5% rate seen in adults aged 18–45 years. Most reactogenicity was mild to moderate, and there were no vaccine-related serious AEs. The complete dose was consumed in 95.3% and 98.1% of vaccine and placebo recipients, respectively. PXVX0200 appears safe, immunogenic, and well tolerated in children and adolescents aged 6–17 years.

## INTRODUCTION

Cholera is an acute enteric infection caused by the ingestion of water or food containing the bacterium *Vibrio cholerae*.^1^ It principally occurs in countries with insufficient access to safe water and proper sanitation, with even more dramatic impact in areas where basic environmental infrastructures are disrupted or have been destroyed.^2^ Cholera is characterized in its most severe form (cholera gravis) by a sudden onset of acute electrolyte-rich watery diarrhea that can lead to severe dehydration and death, an outcome seen most commonly in children younger than 5 years.^1^ The very short incubation period, several hours to 5 days, enhances the potentially explosive pattern of outbreaks.

The current worldwide cholera pandemic has been ongoing since 1961 and is caused by *V. cholerae* O1 El Tor. Cholera occurs in an endemic form in many developing countries and also in explosive outbreaks, as seen in South America in 1991, Haiti in 2010, and Yemen in 2016.^2–4^ It is estimated that 1.3–4.0 million cholera cases, with 21,000–143,000 deaths, occur each year worldwide.^5^ Cholera also represents a risk to travelers to countries with endemic or epidemic cholera.^6–8^ The persistence of cholera in many countries in Asia and Africa, the appearance of particularly severe clinical disease due to El Tor strains expressing classical biotype cholera toxin, and the increasing prevalence of antimicrobial resistance make the control of cholera a high public health priority.^9^

Serum vibriocidal antibodies (SVAs) produced by natural or experimental *V. cholerae* infection correlate with protection against cholera.^10,11^ Experimental (challenge) infection in adults resulted in protective immunity against rechallenge with both homologous and heterologous strains that lasted for at least 3 years.^12^ This led to studies of a number of candidate deletion mutants of classical and El Tor *V. cholerae* O1 designed to produce similar immunity and resulted in the live, attenuated *V. cholerae* strain CVD 103-HgR as a safe and effective oral vaccine for the prevention of cholera.^13^ Clinical trial experience with CVD 103-HgR included administration to more than 27,000 adults and children as young as 3 months of age.^14–23^ CVD 103-HgR was licensed in several countries ex-United States under the trade names Orochol, Orochol E, and Mutachol, and more than 500,000 commercial doses of CVD 103-HgR vaccine were sold with an indication in travelers aged 2 years or older.^24^ Production was discontinued in 2001 for commercial reasons. CVD 103-HgR was acquired by PaxVax in 2009 and was redeveloped under the research name PXVX0200.

The safety, immunogenicity, and efficacy of PXVX0200 in adults were established in four randomized, double-blind, placebo-controlled, multicenter clinical trials. A phase 1 trial demonstrated that PXVX0200 was well tolerated with a SVA seroconversion rate of 89%.^25^ A phase 3 cholera challenge trial of PXVX0200 demonstrated 90% protective efficacy at 10 days and 80% at 3 months versus placebo following ingestion of 1 × 10^5^ wild-type *V. cholerae* O1 El Tor Inaba strain N16961, and also established SVA seroconversion as a correlate of protection against cholera diarrhea.^26,27^ A large phase 3 lot consistency study in 3,146 adults aged 18–45 years demonstrated 93.5% vibriocidal seroconversion 10 days after vaccination with PXVX0200 and further documented safety, whereas another phase 3 study in 398 volunteers aged 46–64 years demonstrated the immunogenicity and safety of PXVX0200 in older adults.^28,29^ As a result of these trials, PXVX0200 was approved by the United States Food and Drug Administration (FDA) in 2016 under the trade name Vaxchora^®^ (PaxVax, Inc., Redwood City, CA) for use in adults aged 18 through 64 years traveling to cholera-affected areas.

Previous studies in children in developing countries using a single dose of the CVD 103-HgR vaccine strain showed the vaccine was well tolerated and suggested that the immune response, as measured by SVA seroconversion rates and geometric mean titers (GMTs), was lower than the response in adults.^14–24^ Immune responses to oral vaccines may be impacted in infants and children in cholera-endemic countries because of natural exposure to infections, age-related differences in immune function, breastfeeding, and nutritional status.^30–32^ Although Orochol was used in children in Europe, Canada, and Australia, there are no published studies of the serological response rates in children in developed countries. Because SVA seroconversion following PXVX0200 vaccination was a strong correlate of protection in the adult challenge study, the FDA accepted that this measure could be used to bridge immunogenicity and presume efficacy in a pediatric population in an industrialized country.^13^ Therefore, this phase 4 study was performed to assess the safety, immunogenicity, and tolerability of a single oral dose of PXVX0200 in children and adolescents in a developed country and to bridge immunogenicity in children and adolescents to adults aged 18–45 years from the lot consistency study.^28^

## METHODS

### Study design.

This was a multicenter phase 4 randomized, double-blind, placebo-controlled trial designed to assess PXVX0200 immunogenicity, safety, palatability, and acceptability in children and adolescents aged 2–17 years. The study was performed at seven U.S. sites from November 2017 through May 2018 in children aged 6–17 years and is ongoing in children aged 2–5 years. Results for children aged 6–17 years are presented here. The study included a screening period of 30 days and a blinding period from day 1 to day 181. Two age cohorts, 12–17 years (cohort 1) and 6–11 years (cohort 2) were enrolled concurrently. Study protocols were reviewed by the institutional review board (IRB) at each site, and informed consent was obtained before screening from each participant’s parent or legal guardian. Participants in cohort 1 provided written assent, whereas participants in cohort 2 provided written assent as per each IRB’s recommendation. The study included healthy children aged 6–17 years without a significant medical history or physical examination findings at screening, as previously described in the adult lot consistency study.^28^ Travel was not restricted during the study. In female participants of childbearing potential, a urine pregnancy test was performed at screening and before vaccine administration. Within each cohort, eligible study participants were randomized 6:1 to receive either a single dose of PXVX0200 or a physiologic saline placebo.

Solicited reactogenicity signs and symptoms included daily temperature, presence of tiredness, headache, lack of appetite, nausea and vomiting, diarrhea, and abdominal pain on study days 1–8 collected using a memory aid (diary card). On day 11, blinded study staff reviewed the signs and symptoms recorded in the memory aid with each participant and parent/guardian to confirm the severity and relationship to study vaccine and action taken (Supplemental Table 1). Unsolicited reactogenicity signs and symptoms were recorded as adverse events (AEs) and were monitored through day 29, whereas serious AEs (SAEs) were monitored and collected through day 181. No efficacy assessments were performed in the study. For serum antibody assays, blood was collected from all participants on days 1, 11, and 29 (±2) with additional blood draws on days 91 (±7) and 181 (±7) in cohort 1.

A Safety Monitoring Committee (SMC), composed of two independent physicians and one independent statistician, conducted periodic reviews of all available, interim, blinded safety data every 6 months. Enrollment was to be stopped, and study medical monitor and SMC review performed before study restart for any death or SAE experienced by a participant, regardless of causality, or any severe or greater AE assessed as possibly or probably related to the study vaccine.

### Vaccine.

Lyophilized PXVX0200 sachets stored at −20°C were reconstituted in a sodium bicarbonate buffer, made using 100 mL of purified bottled water and single-dose buffer sachets containing 2.5 g NaHCO3, 1.5 g ascorbic acid, and 0.2 g lactose. At reconstitution, the single-dose PXVX0200 solution contained 1 × 10^9^ CFU of live-attenuated *V. cholerae*. One vaccine lot, P700.610.00–7000005, was used in the study. Placebo was 100 mL of physiological (0.9%) saline. Study vaccine was prepared and dispensed by an unblinded pharmacist to an unblinded dose administrator, and study product was consumed within 15 minutes of reconstitution. Participants received nothing by mouth for 60 minutes before and after vaccine administration. To maintain blinding, vaccine and placebo were dispensed in opaque cups and the unblinded pharmacist and dose administrator were not involved in postvaccination assessments and evaluations of PXVX0200 reactogenicity, AEs, acceptability, and palatability. The other study site personnel, the participants, their parents or guardians, and sponsor personnel did not know the participants’ treatment assignments. The unblinded dose administrator had the option to add PureVia Stevia sweetener to the oral solution at the request of the parent and/or participant. The sweetener was intended to increase the palatability of the oral solution for pediatric study participants and to increase the likelihood that each participant would consume the full dose. Participants were monitored for acute reactions for 30 minutes after vaccination and were asked not to discuss the taste of the product with study staff or other study participants, as appropriate for age and cognitive level. Clinically significant findings immediately after vaccination were recorded as AEs. After completion of the 30-minute evaluation period, palatability was assessed by the participant and reviewed by blinded study staff using a 5-point hedonic scale.^33^ Blinding was maintained for each participant until they completed their day 181 assessment.

### Immunology.

To determine immunogenicity, titers of classic Inaba SVAs were measured at baseline before vaccine administration (day 1) and 10 and 28 days after vaccination (days 11 and 29) in all participants and also 90 and 180 days after vaccination (days 91 and 181) in cohort 1. These assays were performed at Q2 Solutions, San Juan Capistrano, CA, using assays with previously described methods transferred from the Applied Immunology Unit at the Center for Vaccine Development, University of Maryland, Baltimore.^34^

### Statistical analysis.

#### Sample size determination.

The trial was designed to enable the independent evaluation of the immunogenicity of PXVX0200 in three separate age cohorts for two co-primary objectives: non-inferiority to adults in the rate of seroconversion and a minimum seroconversion rate of 70%. The statistical CIs used for assessing the two primary objectives were adjusted for multiplicity by allocating 2/3 of the type I error or alpha (0.033) to the non-inferiority objective and the remaining 1/3 of the alpha (0.0167) to the adequacy of the seroconversion rate. As a result, rather than the typical 95% CIs, two-sided 96.7% CIs and 98.3% CIs for the non-inferiority and adequacy endpoints, respectively, were used. The separate age cohorts were then tested sequentially within each objective to maintain the type 1 error across cohorts. The older age cohort (12–17 years) was assessed first, and, only if that age cohort achieved the success criterion, could the next younger age cohort be assessed.

To meet the co-primary endpoints, the lower bound of the two-sided 96.7% CIs on the difference in seroconversion rates between children and adults aged 18–45 years was required to be greater than −10 percentage points and the lower bound of the two-sided 98.3% CI on the proportion of vaccinees who seroconverted between days 1 and 11 was required to be equal or exceed 70%.

Assuming that the true seroconversion rate was 92.4% or higher, the sample size of 143 evaluable vaccinees for an age cohort afforded 93.3% power to demonstrate that the seroconversion rate within the age cohort was non-inferior to the 93.5% rate observed in the 2,687 adult participants assessed in the lot consistency trial.^28^ Assuming that the rates in the three age cohorts are independent of one another, the overall power for demonstrating the non-inferiority of all three age cohorts [(93.3%)^3^] is 81%.

Under the same assumptions as earlier—in particular, assuming the true seroconversion rate within an age cohort is at least 92.4%–143 evaluable vaccinees provided greater than 99.9% power to establish that the lower bound on the cohort-specific rate is at least 70%. Power would still be greater than 99.9% when requiring the lower bound on all three cohorts to be 70% or greater.

The overall power of meeting both primary objectives in all three age cohorts can be approximated by multiplying the power of meeting the non-inferiority objective by the power of meeting the 70% lower bound objective: 81% × 99.9% ≈ 81%.

### Immunogenicity.

The primary endpoint was the proportion of participants achieving seroconversion, defined as a 4-fold or greater rise over day 1 SVAs, at day 11 after one dose of PXVX0200 in the immunogenicity evaluable population (IEP), which included the set of participants who received vaccination, consumed at least 80% of the study treatment, had evaluable results from both day 1 and day 11, and had no major protocol violations that would affect immunogenicity. The proportion of seroconverters was called the seroconversion rate. Each rate was accompanied by a CI calculated using the Wilson method, with the size of the CI adjusted for the type of comparison needed (the placebo comparison used a 95% CI).^35^ Cumulative seroconversion through a particular visit was defined as the occurrence of seroconversion at or before that visit. For each cohort, the proportion of vaccinated participants who experienced a 4-fold or greater increase in serum vibriocidal titer between day 1 and day 11 was calculated and the 98.3% CIs on this proportion of seroconverters was calculated. The seroconversion rate for each cohort was compared with the seroconversion rate for the bridging population, vaccinees aged 18–45 years who participated in the lot consistency study, by calculating the difference between the two rates and computing a 96.7% CI for this difference.^28^

#### Additional immunogenicity objectives.

The point estimate of the seroconversion rate for vaccinees as well as the cumulative seroconversion rate, and 95% CIs were also computed at the time points on days 29, 91, and 181 (day 29 only for cohort 2). The GMT and 95% CIs were computed for days 1, 11, and 29 (both cohorts), and for days 91 and 181 (cohort 1 only), and the geometric mean fold increase (GMFI) in titer in each treatment group was calculated from day 1 for each of the time point. GMTs and associated 95% CIs were calculated by first constructing the mean and CI on log-transformed data and then back-transforming to the original data space.

#### Acceptability and palatability.

Acceptability was evaluated by assessing the percent of participants in each age cohort able to complete the dosing according to the protocol. This was defined as the entire volume of dose being consumed within 15 minutes after reconstitution. Palatability of vaccine was assessed by the participants using a 5-point hedonic scale.^33^

#### Reactogenicity and safety.

Solicited reactogenicity was summarized by the frequency in each treatment group of each of the solicited reactogenicity signs or symptoms as well as summaries by severity and treatment group and were compared using Fisher’s exact tests (Supplemental Table 1). Unsolicited treatment emergent AEs and SAEs were summarized by system organ class and preferred term, by severity and by count and percent for PXVX0200 and placebo groups, by relationship to study vaccine, by age cohort, and overall.

## RESULTS

### Demographics.

**Figure 1.**
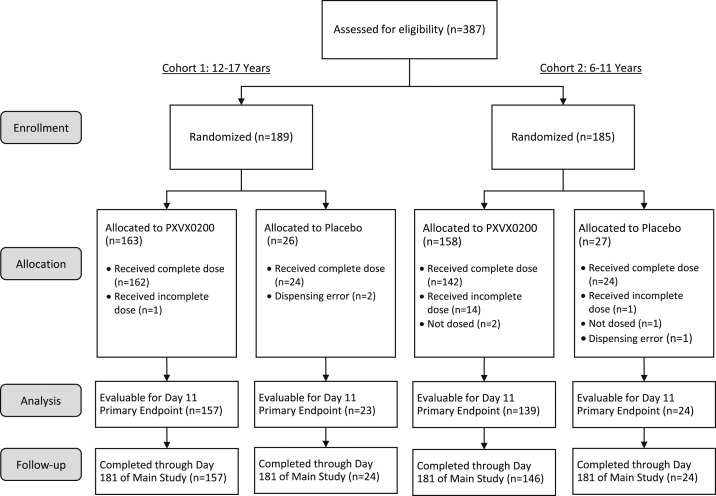
Participant disposition. Consolidated Standards of Reporting Tools (CONSORT) diagram for pediatric trial: Reasons for withdrawal were as follows: 10 participants withdrew consent, 10 were lost to follow-up, two were noncompliant with the protocol, and one ineligible participant was accidentally randomized and withdrawn.

A total of 387 participants between the ages of 6 and 17 years were screened, and 374 were randomized across seven study sites, with 40 or more subjects enrolled at six sites. In cohort 1 (aged 12–17 years), 197 participants were screened, 189 were randomized, and 181 (95.8%) completed the study through the day 181 visit. In cohort 2 (aged 6–11 years), 190 participants were screened, 185 were randomized, and 170 (91.9%) completed the study through the day 181 visit (Figure 1). There were six participants who were randomized and had duplicate enrollment in error. These subjects were excluded from the immunogenicity evaluations but were included in the safety population. Participant characteristics for the study groups for randomized participants are presented in Table 1. Other than age, the demographic and other baseline characteristics of the treatment groups and cohorts were similar.

**Table 1:** Participant demographics by age, cohort, and treatment group

Baseline characteristic	Cohort 1 (aged 12–17 years)		Cohort 2 (aged 6–11 years)		Overall (aged 6–17 years)		Total (*N* = 374)
	PXVX0200 (*N* = 163)	Placebo (*N* = 26)	PXVX0200 (*N* = 158)	Placebo (*N* = 27)	PXVX0200 (*N* = 321)	Placebo (*N* = 53)	
Age (years)							
Mean ± SD	14.4 ± 1.7	14.3 ± 1.7	8.6 ± 1.8	8.7 ± 1.5	11.5 ± 3.4	11.4 ± 3.2	11.5 ± 3.4
Median (min–max)	14.0 (12–17)	15.0 (12–17)	9.0 (6–11)	9.0 (6–11)	12.0 (6–17)	11.0 (6–17)	12.0 (6–17)
Gender							
Male, *n* (%)	88 (54.0)	14 (53.8)	77 (48.7)	17 (63.0)	165 (51.4)	31 (58.5)	196 (52.4)
Female, *n* (%)	75 (46.0)	12 (46.2)	81 (51.3)	10 (37.0)	156 (48.6)	22 (41.5)	178 (47.6)
Race							
American Indian or Alaskan Native, *n* (%)	0	1 (3.8)	0	1 (3.7)	0	2 (3.8)	2 (0.5)
Asian, *n* (%)	1 (0.6)	0	4 (2.5)	0	5 (1.6)	0	5 (1.3)
Native Hawaiian or other Pacific Islander	0	0	0	0	0	0	0
Black or African American, *n* (%)	28 (17.2)	4 (15.4)	53 (33.5)	5 (18.5)	81 (25.2)	9 (17.0)	90 (24.1)
White, *n* (%)	121 (74.2)	21 (80.8)	86 (54.4)	18 (66.7)	207 (64.5)	39 (73.6)	246 (65.8)
Multiple, *n* (%)	13 (8.0)	0	15 (9.5)	3 (11.1)	28 (8.7)	3 (5.7)	31 (8.3)
Other	0	0	0	0	0	0	0
Ethnicity							
Hispanic or Latino, *n* (%)	18 (11.0)	7 (26.9)	11 (7.0)	2 (7.4)	29 (9.0)	9 (17.0)	38 (10.2)
Not Hispanic or Latino, *n* (%)	145 (89.0)	19 (73.1)	147 (93.0)	25 (92.6)	292 (91.0)	44 (83.0)	336 (89.8)
BMI (kg/m^2^)							
Mean (SD)	23.6 (5.7)	25.0 (4.8)	18.7 (5.2)	17.5 (3.1)	21.2 (6.0)	21.2 (5.5)	21.2 (5.9)
Median (min–max)	22.5 (14.3–45.1)	24.2 (17.8–36.6)	17.1 (11.7–44.6)	16.9 (13.0–26.2)	20.4 (11.7–45.1)	20.0 (13.0–36.6)	20.3 (11.7–45.1)

BMI = body mass index.

### Immunogenicity.

#### Non-inferiority.

The day 11 seroconversion rates of the PXVX0200 participants in the IEP group compared with those in the adult bridging population from the lot consistency study are shown in Table 2. For cohort 1, both primary objectives were met. Cohort 1 was non-inferior to the adult bridging population, with the lower limit of the 96.7% CIs on the difference between the groups exceeding the required −10 percentage points (difference = +5.8%; 96.7% CI: [2.4, 7.1]). There was also a statistically significant increase in the percentage of participants who seroconverted compared with the adult bridging population (*P* < 0.01). For the second co-primary endpoint, the cohort 1 participants had a 99.4% day 11 seroconversion rate and the 98.3% CI was [95.4, 99.9%], which well surpassed the protocol required lower confidence limit on seroconversion of 70%.

**Table 2:** Comparison of seroconversion rates at day 11 visit by age-group compared with the adult bridging population

Seroconversion	Adults (aged 18–45 years)	Cohort 1 (aged 12–17 years)	Cohort 2 (aged 6–11 years)	Overall (aged 6–17 years)
	PXVX0200	PXVX0200	PXVX0200	PXVX0200
Day 11 visit				
*N* analyzable	2,687	157	139	296
*N* (%) seroconverted [98.3% CI]	2,513 (93.5) [92.3%, 94.6%]	156 (99.4) [95.4%, 99.9%]	136 (97.8) [92.5%, 99.4%]	292 (98.6) [95.9%, 99.6%]
Difference (Cohort *minus* adults)	–	5.8%	4.3%	5.1%
96.7% CI on % difference	–	[2.4%, 7.1%]	[-0.3%, 6.2%]	[2.6%, 6.5%]
*P*-value	–	0.0009	0.0455	< 0.0001

For cohort 2, both co-primary objectives were also met. Cohort 2 was non-inferior to the adult bridging population, with the lower limit of the 96.7% CI on the difference between the groups exceeding the required −10 percentage points (difference = +4.3%; 96.7% CI: [−0.3, 6.2]). For the second co-primary objective, the cohort 2 participants had a 97.8% day 11 seroconversion rate and the 98.3% CI was [92.5, 99.4%], which was well greater than the protocol required lower limit of 70% with seroconversion.

The overall seroconversion rate was 98.6% among the pediatric PXVX0200 recipients (98.3% CI: [95.9%, 99.6%]) and was significantly higher than the adult seroconversion rate of 93.5% (*P* < 0.01).

The primary dataset used for analysis was the IEP. However, a robustness analysis based on the modified intent-to-treat population, which only excluded participants who were missing day 1 or day 11 SVA results, was included to determine the degree to which protocol deviations may have affected the results. The overall conclusion of non-inferiority to the adults and seroconversion was unaffected by the choice of analysis population.

#### Additional immunogenicity objectives.

The cumulative seroconversion rates by the treatment group and cohort are shown in Table 3. The cumulative seroconversion rate at day 29 for PXVX0200 recipients in cohort 1 was 100% versus 0% for the placebo recipients (*P* < 0.0001). The cumulative seroconversion rate at day 29 for cohort 2 was 97.8% versus 8.3% for the placebo recipients (*P* < 0.0001). The overall day 29 cumulative seroconversion rate was 99.0% for PXVX0200 recipients versus 4.3% for placebo recipients (*P* < 0.0001).

**Table 3:** Cumulative seroconversion rate by treatment group and cohort

Seroconversion	Cohort 1 (aged 12–17 years)		Cohort 2 (aged 6–11 years)		Overall (aged 6–17 years)	
	PXVX0200 (*N* = 157)	Placebo (*N* = 23)	PXVX0200 (*N* = 139)	Placebo (*N* = 24)	PXVX0200 (*N* = 296)	Placebo (*N* = 47)
Day 11 visit						
*N* analyzable	157	23	139	24	296	47
*N* (%) seroconverted (95% CI)	156 (99.4%)* [96.5%, 99.9%]	0 [0%, 14.3%]	136 (97.8%)* [93.8%, 99.3%]	1 (4.2%) [0.7%, 20.2%]	292 (98.6%)* [96.6%, 99.5%]	1 (2.1%) [0.4%, 11.1%]
Day 29 visit						
*N* analyzable	157	23	139	24	296	47
*N* (%) seroconverted (95% CI)	157 (100%)* [97.6%, 100%]	0 [0%, 14.3%]	136 (97.8%)* [93.8%, 99.3%]	2 (8.3%) [2.3%, 25.8%]	293 (99.0%)* [97.1%, 99.7%]	2 (4.3%) [1.2%, 14.2%]
Day 91 visit						
*N* analyzable	157	23	–	–	–	–
*N* (%) seroconverted (95% CI)	157 (100%)* [97.6%, 100%]	0 [0%, 14.3%]	–	–	–	–
Day 181 visit						
*N* analyzable	157	23	–	–	–	–
*N* (%) seroconverted (95% CI)	157 (100%)* [97.6%, 100%]	0 [0%, 14.3%]	–	–	–	–

* *P* < 0.0001 from Fisher’s Exact test of equality of seroconversion between the PXVX0200 and placebo.

**Figure 2.**
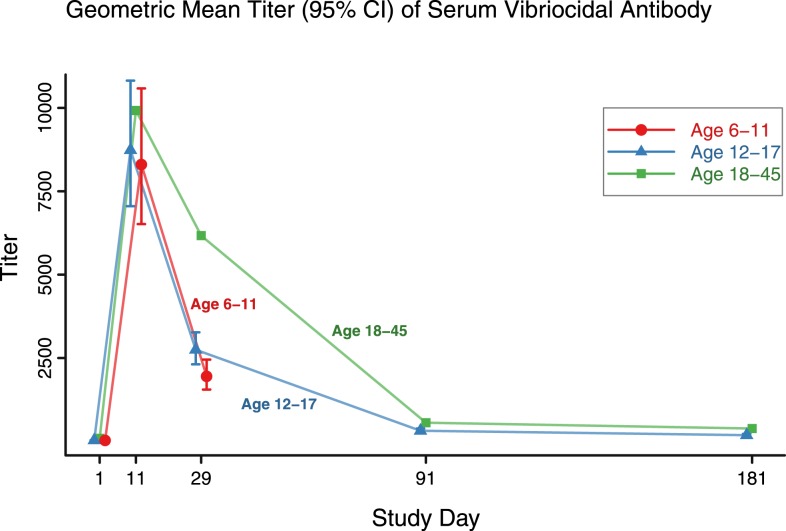
Geometric mean titer evels by age cohort and treatment group. Cohort 2 (aged 6–11 years) titers followed to day 29 only. This figure appears in color at www.ajtmh.org.

The GMT of serum vibriocidal titers for PXVX0200 recipients in cohort 1 peaked at 8,735 on day 11 and decreased through day 181 at which point titers remained significantly greater than placebo (Table 4). The GMT of serum vibriocidal titers for PXVX0200 recipients in cohort 2 peaked at 8,305 on day 11 and decreased to 1952 at day 29. In both cohorts, all comparisons of PXVX0200 to placebo were highly significant (*P* < 0.0001). Figure 2 shows GMTs over time for vaccine recipients along with the analogous measurements from the bridging population in the adult lot consistency study.

**Table 4:** Geometric mean titers (GMT) against classical Inaba *Vibrio cholerae*, all time points by age-group and cohort, immunogenicity evaluable population

Geometric mean	Cohort 1 (aged 12–17 years)		Cohort 2 (aged 6–11 years)		Overall (aged 6–17 years)	
	PXVX0200 (*N* = 157)	Placebo (*N* = 23)	PXVX0200 (*N* = 139)	Placebo (*N* = 24)	PXVX0200 (*N* = 296)	Placebo (*N* = 47)
Day 1 visit						
*N* analyzable	157	23	139	24	296	47
GMT	32	44	32	36	32	39
(95% CI)	[28, 37]	[28, 70]	[28, 36]	[24, 52]	[29, 35]	[30, 53]
Min, max	20, 640	20, 1,280	20, 1,280	20, 640	20, 1,280	20, 1,280
Day 11 visit						
*N* analyzable	157	23	139	24	296	47
GMT	8,735*	41	8,305*	40	8,531*	41
(95% CI)	[7,053, 10,819]	[26, 65]	[6,516, 10,586]	[23, 70]	[7,270, 10,009, ]	[29, 58]
Min, max	20, 81,920	20, 1,280	40, 163,840	20, 5,120	20, 163,840	20, 5,120
Day 29 visit						
*N* analyzable	156	23	138	23	294	46
GMT	2,748*	43	1,952*	40	2,341*	41
(95% CI)	[2,311, 3,270]	[27, 67]	[1,554, 2,452]	[22, 72]	[2,031, 2,697]	[29, 59]
Min, max	320, 20,480	20, 640	40, 40,960	20, 5,120	40, 40,960	20, 5,120
Day 91 visit						
*N* analyzable	153	23	–	–	–	–
GMT	319*	43	–	–	–	–
(95% CI)	[263, 386]	[29, 63]	–	–	–	–
Min, max	20, 5,120	20, 640	–	–	–	–
Day 181 visit						
*N* analyzable	151	21	–	–	–	–
GMT	186*	39	–	–	–	–
(95% CI)	[154, 225]	[26, 58]	–	–	–	–
Min, max	20, 5,120	20, 320	–	–	–	–

* *P* < 0.0001; *P*-values are based on *t*-statistics assuming normal distribution of the log titer.

The results for GMFI in SVA titers are shown in Table 5. The GMFI of vibriocidal antibodies for cohort 1 PXVX0200 participants was 272 times baseline value at day 11 and 85 times baseline at day 29. The GMFI of vibriocidal antibodies for cohort 2 PXVX0200 participants was 264 times baseline at day 11 and 62 times baseline at day 29. As with the GMTs, the comparisons of PXVX0200 to placebo for mean fold increase were highly significant for both cohorts at all visits (*P* < 0.0001).

**Table 5:** Geometric mean fold increase against classical Inaba *Vibrio cholerae* all time points by age group and cohort—immunogenicity evaluable population

Fold increase	Cohort 1 (aged 12–17 years)		Cohort 2 (aged 6–11 years)		Overall (aged 6–17 years)	
	PXVX0200 (*N* = 160)	Placebo (*N* = 24)	PXVX0200 (*N* = 148)	Placebo (*N* = 24)	PXVX0200 (*N* = 308)	Placebo (*N* = 48)
Day 11 visit						
*N* analyzable	157	23	139	24	296	47
Mean fold increase	272*	1	264*	1	268*	1
(95% CI)	[222, 334]	[1, 1]	[204, 341]	[1, 2]	[229, 315]	[1, 1]
Min, max	1, 4,096	0.3, 2	1, 4,096	1, 256	1, 4,096	0.3, 256
Day 29 visit						
*N* analyzable	156	23	138	23	294	46
Mean fold increase	85*	1	62*	1	73*	1
(95% CI)	[72, 102]	[1, 1]	[49, 78]	[1,2]	[64, 85]	[1, 1]
Min, max	8, 1,024	0.3, 2	1, 1,024	1, 32	1, 1,024	0.3, 32
Day 91 visit						
*N* analyzable	153	23	–	–	–	–
Mean fold increase	10*	1	–	–	–	–
(95% CI)	[8, 12]	[1, 1]	–	–	–	–
Min, max	1, 256	0.1, 2	–	–	–	–
Day 181 visit						
*N* analyzable	151	21	–	–	–	–
Mean fold increase	6*	1	–	–	–	–
(95% CI)	[5, 7]	[1, 1]	–	–	–	–
Min, max	1, 128	0.3, 2	–	–	–	–

* *P* < 0.0001; *P*-values are based on *t*-statistics assuming normal distribution of the log titer.

#### Acceptability.

In cohort 1, 99.4% of PXVX0200 recipients and 100% of placebo recipients were completely dosed with the correct respective oral solutions (Table 6). In cohort 2, 91.0% of PXVX0200 recipients and 96.2% of placebo recipients were completely dosed with the correct respective oral solutions.

**Table 6:** Vaccine acceptability by age cohort and randomized treatment group

Dosing	Cohort 1 (aged 12–17 years)		Cohort 2 (aged 6–11 years)		Overall (aged 6–17 years)	
	PXVX0200 (*N* = 163)	Placebo (*N* = 26)	PXVX0200 (*N* = 156)	Placebo (*N* = 26)	PXVX0200 (*N* = 319)	Placebo (*N* = 52)
Complete dose consumed						
Yes, *n* (%)	162 (99.4)	26 (100)	142 (91.0)	25 (96.2)	304 (95.3)	51 (98.1)
No, *n* (%)	1 (0.6)	0	14 (9.0)	1 (3.8)	15 (4.7)	1 (1.9)

#### Palatability.

Results of palatability testing are presented in Table 7. The palatability assessments did not differ significantly between the vaccine and placebo groups within each cohort. Issues with palatability possibly contributed to the percentage of participants who did not consume a complete dose of their respective randomized treatment in cohort 2. Of cohort 2 participants with unacceptable dosing, palatability was reported as “very bad” by 80% of PVXV0200 recipients and by one placebo recipient. Addition of PureVia Stevia sweetener did not improve participants’ opinions regarding treatment palatability.

**Table 7:** Palatability by age cohort and treatment group in subjects who received treatment

Response (*n* [%])	Cohort 1 (aged 12–17 years)		Cohort 2 (aged 6–11 years)		Overall (aged 6–17 years)	
	PXVX0200 (N = 163)	Placebo (N = 26)	PXVX0200 (N = 156)	Placebo (N = 26)	PXVX0200 (N = 319)	Placebo (N = 52)
Palatability rating						
Very bad	12 (7.4)	1 (3.8)	34 (21.8)	6 (23.1)	46 (14.4)	7 (13.5)
Bad	50 (30.7)	7 (26.9)	30 (19.2)	6 (23.1)	80 (25.1)	13 (25.0)
Neutral	64 (39.3)	10 (38.5)	37 (23.7)	10 (38.5)	101 (31.7)	20 (38.5)
Good	29 (17.8)	6 (23.1)	27 (17.3)	1 (3.8)	56 (17.6)	7 (13.5)
Very good	8 (4.9)	2 (7.7)	28 (17.9)	3 (11.5)	36 (11.3)	5 (9.6)
Sweetener added						
Yes	144 (88.3)	22 (84.6)	149 (95.5)	25 (96.2)	293 (91.8)	47 (90.4)

#### Reactogenicity and safety.

The frequency and severity of solicited reactogenicity signs and symptoms for PXVX0200 and placebo recipients are reported in Table 8. Reactogenicity was more common in cohort 1 than in cohort 2. The number of participants with any solicited AE was similar between both treatment groups, and the median duration of symptoms was 3 days for cohort 1 and 2 days for cohort 2. Among solicited AEs, the most frequent across both cohorts and treatment groups were tiredness, headache, and abdominal pain. Diarrhea was reported in 6/165 (3.6%) of PXVX0200 recipients in cohort 1 and was not reported by any study participants in cohort 2. Abdominal pain and vomiting were more frequently reported by PXVX0200 recipients than by placebo recipients, but these differences were not statistically significant. Across both cohorts, there were four participants who reported severe solicited AEs that were considered treatment related, including one with nausea, vomiting, and diarrhea; one with abdominal pain and diarrhea; and one each with fever or diarrhea only. All of these participants were PXVX0200 recipients. Study enrollment was stopped five times for severe solicited reactogenicity signs/symptoms, four considered possibly related to study product by the investigator, one severe fever considered unrelated, and for one unrelated SAE. In each case, SMC review was performed and the study restarted without modification. Unsolicited AEs were reported by 24.5% of PXVX0200 recipients and 32.7% of placebo recipients, and most were considered unrelated to study treatment. There were no deaths, treatment-related SAEs, or discontinuations due to AEs in either age cohort or treatment group. There was one unrelated SAE, a lower limb fracture, reported in a PXVX0200 recipient.

**Table 8:** Frequency and severity of solicited reactogenicity reported

Solicited event [n (%)]	Cohort 1 (aged 12–17 years)		Cohort 2 (aged 6–11 years)		Overall (aged 6–17 years)	
	PXVX0200 (*N* = 165)	Placebo (*N* = 24)	PXVX0200 (*N* = 157)	Placebo (*N* = 25)	PXVX0200 (*N* = 322)	Placebo (*N* = 49)
Any event	113 (68.5)	16 (66.7)	86 (54.8)	13 (52.0)	199 (61.8)	29 (59.2)
Mild/moderate	109 (66.1)	15 (62.5)	81 (51.6)	13 (52.0)	190 (59.0)	28 (57.1)
Severe	4 (2.4)*	1 (4.2)	5 (3.2)	0	9 (2.8)*	1 (2.0)
Tiredness	67 (40.6)	9 (37.5)	55 (35.0)	8 (32.0)	122 (37.9)	17 (34.7)
Mild/moderate	66 (40.0)	8 (33.3)	54 (34.4)	8 (32.0)	120 (37.2)	16 (32.6)
Severe	1 (0.6)*	1 (4.2)	1 (0.6)	0	2 (0.6)*	1 (2.0)
Headache	74 (44.8)	11 (45.8)	41 (26.1)	6 (24.0)	115 (35.7)	17 (34.7)
Mild/moderate	73 (44.2)	11 (45.8)	39 (24.8)	6 (24.0)	112 (34.8)	17 (34.7)
Severe	1 (0.6)	0	2 (1.3)	0	3 (0.9)	0
Abdominal pain	62 (37.6)	4 (16.7)	43 (27.4)	6 (24.0)	105 (32.6)	10 (20.4)
Mild/moderate	61 (37.0)	4 (16.7)	43 (27.4)	6 (24.0)	104 (32.3)	10 (20.4)
Severe	1 (0.6)	0	0	0	1 (0.3)	0
Lack of appetite	48 (29.1)	3 (12.5)	24 (15.3)	5 (20.0)	72 (22.4)	8 (16.3)
Mild/moderate	48 (29.1)	3 (12.5)	23 (14.6)	5 (20.0)	71 (22.0)	8 (16.3)
Severe	0	0	1 (0.6)	0	1 (0.3)	0
Nausea	37 (22.4)	6 (25.0)	22 (14.0)	4 (16.0)	59 (18.3)	10 (20.4)
Mild/moderate	36 (21.8)	6 (25.0)	22 (14.0)	4 (16.0)	58 (18.0)	10 (20.4)
Severe	1 (0.6)	0	0	0	1 (0.3)	0
Vomiting	9 (5.5)	0	7 (4.5)	0	16 (5.0)	0
Mild/moderate	8 (4.8)	0	7 (4.5)	0	15 (4.7)	0
Severe	1 (0.6)	0	0	0	1 (0.3)	0
Fever	3 (1.8)	0	5 (3.2)	1 (4.0)	8 (2.5)	1 (2.0)
Mild/moderate	2 (1.2)	0	1 (0.6)	1 (4.0)	3 (0.9)	1 (2.0)
Severe	1 (0.6)	0	4 (2.5)	0	5 (1.6)	0
Diarrhea	6 (3.6)	1 (4.2)	0	0	6 (1.9)	1 (2.0)
Mild/moderate	3 (1.8)	1 (4.2)	0	0	3 (0.9)	1 (2.0)
Severe	3 (1.8)	0	0	0	3 (0.9)	0

No comparison between PXVX0200 and placebo groups was statistically significant.

* Includes one case of potentially life-threatening tiredness.

## DISCUSSION

Cholera is a potentially lethal infection that occurs throughout the world in both endemic forms and as explosive outbreaks in “virgin soil” populations.^2,3^ It also represents a threat to both children and adults from developed countries who travel to cholera-endemic areas and can cause severe and fatal disease.^4,6–8,36,37^ A single-dose vaccine that could provide rapid protection in all age-groups to both travelers to and inhabitants of countries at risk for cholera would be ideal.

This study documents the safety, immunogenicity, and tolerability of a single-dose, live oral cholera vaccine PXVX0200 in children and adolescents aged 6–17 years in a developed country. Because *V. cholerae* challenge studies are not feasible in a pediatric population, we undertook an immunologic bridging study with SVA seroconversion as a correlate of protection. Bridging studies are performed to extrapolate efficacy for vaccines for which a correlate of protection exists and SVA seroconversion, defined as a ≥ 4-fold rise in vibriocidal antibody, was shown to be a strong serological correlate of protection in the PXVX0200 challenge study.^26,27,38^ In the current trial, SVA seroconversion 10 days after vaccination occurred in 99.4% of recipients aged 12–17 years (cohort 1) and in 97.8% of vaccine recipients aged 6–11 years (cohort 2) versus 0% and 4.2%, respectively, in placebo recipients (*P* < 0.0001). These rates compared favorably with the seroconversion rate of 93.5% seen in the bridging population of adults aged 18–45 years in the phase 3 lot consistency study, and because seroconversion was greater than 70% in each cohort, both co-primary endpoints were met. Of note, although the SVA seroconversion rate was non-inferior in participants aged 6–11 years when compared with adults aged 18–45 years, it was significantly higher than that for adults in participants aged 12–17 years (*P* = 0.0009). Because SVAs have long been known to be a correlate of protection against cholera infection, vaccine recipients aged 6–17 years would be predicted to be protected against cholera-induced diarrhea.^26,27,39,40^ The day 11 SVA seroconversion rates in placebo recipients aged 6–17 years (2.1%) were also similar to those seen in adult placebo recipients aged 18–45 years (4.2%).

Studies of CVD 103-HgR in North American adults also documented protection against diarrhea caused by both the classical and El Tor biotypes and both the Inaba and Ogawa serotypes of *V. cholerae* O1, and protection against these other types of cholera in children and adolescents could possibly be inferred.^41–44^ GMT kinetics in cohort 1 and cohort 2 (Figure 2) were similar to those in adults aged 18–45 years in the lot consistency study.^28^ Thus, analysis of GMTs, geometric fold increase, and cumulative seroconversion rates confirm that PXVX0200 was immunogenic in this pediatric population.

Previous studies of a single dose of the CVD 103-HgR vaccine strain in children in developing countries showed that the vaccine was well tolerated and demonstrated that the immune responses, as measured by SVA seroconversion rates and GMTs, were lower than the responses seen in adults in developed countries, as well as in Thai adults from higher socioeconomic levels.^14–17,19–23,44–47^ As a result, CVD 103-HgR dosages were increased 10-fold to achieve higher rates of seroconversion and the vaccine was marketed in developing countries as the Orochol E formulation which contained 5 × 10^9^ CFU/dose. For example, Suharyono documented SVA seroconversion in 16% of 5- to 9-year-old participants living in squalid conditions in Indonesia immunized with 5 × 10^8^ CFU of CVD 103-HgR and in 75–87% of those immunized with 5 × 10^9^ CFU.^14^ Most of the previous studies of CVD 103-HgR in children in developing countries used a dose of 5 × 10^9^ CFU with seroconversion seen in only 22–78% of vaccine recipients.

Immune responses to oral vaccines, including polio virus, rotavirus, and cholera, may be lower in infants and children in developing countries, and thus higher CVD 103-HgR dosages are required to induce protective antibodies.^30–32^ These reduced immune responses may be due to multiple factors, including malnutrition, maternal undernutrition during pregnancy, small bowel overgrowth with intestinal mucosa damage (chronic environmental enteropathy) from living in fecally contaminated environments, parasitic infections, intestinal enteric virus infections, interference from breast milk antibody, and preexisting immunity due to natural exposure to infection. Chronic environmental enteropathy, also known as tropical enteropathy, a subclinical inflammatory condition of the gut, is associated with changes in the gut microbiota and intestinal barrier function, upregulation of pro-inflammatory cytokines and chemokines, and decreased responsiveness to oral polio and rotavirus vaccines.^32^ A study of small bowel overgrowth documented an association between peak breath hydrogen testing and diminished seroconversion following immunization with CVD 103-HgR.^19^ An efficacy trial of CVD 103-HgR in Indonesia documented seroconversion in 42% of participants with high baseline vibriocidal titers versus 86% in those with low titers, suggesting that some individuals may already be immune to cholera and that their serum titers are not boosted substantially by immunization.^21,31^ Another trial documented increased seroconversion following vaccination with CVD 103-HgR in children with ascariasis treated with albendazole.^22^ None of the conditions associated with decreased seroconversion described here is common in the United States or other developed countries, and it is expected that children from these countries will mount a more substantial immune response to PXVX0200.

The study vaccine was well accepted with 99.4% and 91.0%, respectively, of cohort 1 and cohort 2 PXVX0200 recipients able to complete dosing, suggesting that age influenced the willingness and ability to consume the oral solution. It is likely that palatability impacted the acceptability of the study vaccine because 8/10 (80%) of the cohort 2 PXVX0200 recipients with unacceptable dosing rated palatability as “very bad.” Overall, there was no significant difference in palatability assessments between vaccine and placebo recipients in each cohort. It is notable that in one trial of CVD 103-HgR performed in infants and toddlers in Chile, the seroconversion rate was similar in fully vaccinated participants (66%) and in those who ingested a smaller fraction of the vaccine (63%).^20^ However, these results may not be applicable to the current study, and, in general, the acceptability and palatability of any pediatric oral medication or vaccine are critical to compliance and, thus, its effectiveness.^48,49^ Addition of sweetener did not improve palatability and will not be used in future PXVX0200 pediatric trials.

Other than one small study performed in Austria, there are no previous studies of the safety of CVD 103-HgR in children in developed countries.^18^ The data from this study demonstrated that PXVX0200 is well tolerated in children and adolescents, with a safety profile similar to that seen in adults and also to Orochol, the previously marketed form of CVD 103-HgR.^24–26,28,29^ Solicited reactogenicity was more frequently reported in both groups in cohort 1, consistent with a reporting bias as this older age-group may have been better able to communicate feeling unwell. Abdominal pain and vomiting may have been more common in PXVX0200 recipients although in the vast majority of participants these were mild to moderate in severity. Diarrhea is a potentially important AE with live oral cholera vaccine. However, in this study, it was reported with similar frequency by vaccine and placebo recipients, although severe diarrhea was noted in three (0.9%) PVXV0200 recipients. These are similar to the rates of diarrhea seen in adults in the lot consistency study and in children aged 2–9 years with the previously commercialized form of CVD 103-HgR.^24,28^ There were no differences between vaccine and placebo recipients in the incidence of unsolicited AEs, and there were no vaccine-related SAEs.

Two other cholera vaccines are available outside of the United States, WC-rBS (Dukoral) and bivalent, killed whole-cell vaccine (Shanchol, Euvichol), administered as two or three doses depending on age.^50^ As a single-dose immunization, PXVX0200 could offer a potential advantage in an outbreak setting in a vulnerable pediatric population.

## CONCLUSION

These data demonstrate that PXVX0200 may be a safe, tolerable cholera vaccine option that produces a robust vibriocidal antibody response in children and adolescents aged 6–17 years and would be expected to provide protection against cholera in this pediatric population from developed countries who are at an increased risk of infection when traveling to or residing in at-risk countries.

## Supplemental file

Supplemental materials
