# Supplementary material for: Safety and Immunogenicity of Live Oral Cholera Vaccine CVD 103-HgR in Children and Adolescents Aged 6–17 Years
Source: Am J Trop Med Hyg. 2019 Nov 25;102(1):48–57. doi: 10.4269/ajtmh.19-0241 (PMC6947768; doi:10.4269/ajtmh.19-0241)
Supplement: Supplementary file 1 [file tpmd190241.SD1.docx]

Supplemental Table 1: Toxicity Grading Scale

**Table for Clinical Abnormalities**

| VACCINE REACTION | MILD (Grade 1) | MODERATE (Grade 2) | SEVERE (Grade 3) | POTENTIALLY LIFE THREATENING (Grade 4) |
| --- | --- | --- | --- | --- |
| Abdominal Pain | Mild, no interference with activity | Some interference with activity | Significant, prevents daily activity | ER visit or hospitalization for hypotensive shock |
| Anorexia (Lack of appetite) | No interference with activity | Some interference with activity | Significant, prevents daily activity | ER visit or hospitalization |
| Diarrhea | 4 loose stools /24 hours | 5 loose stools/24 hours | > 6 loose stools/24 hours | ER visit or hospitalization |
| Fatigue (Tiredness) | Mild, no interference with activity | Some interference with activity | Significant, prevents daily activity | ER visit or hospitalization |
| Fever | > 100.4 – 101.1°F (>38.0 –38.4°C) | >101.2 – 102°F (≥38.5 –38.9°C) | > 102.1°F–104°F (≥39°C–40°C) | > 104°F (> 40°C) |
| Headache | Mild, no interference with activity | Some interference with activity | Significant, prevents daily activity | ER visit or hospitalization |
| Myalgia | Mild, no interference with activity | Some interference with activity | Significant, prevents daily activity | ER visit or hospitalization |
| Nausea | Mild, no interference with activity | Some interference with activity | Significant, prevents daily activity | ER visit or hospitalization for hypotensive shock |
| Vomiting | 1–2 episodes/24 hours | > 2 episodes/24 hours | Requires IV hydration | ER visit or hospitalization for hypotensive shock |

When developing this Toxicity Grading Scale, PaxVax referred to the recommendations in the FDA’s Guidance for Industry: Toxicity Grading Scale for Healthy Adult and Adolescent Subjects Enrolled in Preventive Vaccine Clinical Trials ([US FDA 2007](#_Tacket_CO,_Cohen)).

Diarrhea 1-3 Stools/24 hours: Record as “loose stools” on the AE CRF.
